# Supplementary material for: Single‐Cell RNA Sequencing of Peripheral Blood Mononuclear Cells in Patients With Single Ventricle/Hypoplastic Left Heart Syndrome
Source: J Gene Med. 2025 Aug 19;27(8):e70030. doi: 10.1002/jgm.70030 (PMC12365367; doi:10.1002/jgm.70030)
Supplement: Supplementary file 2 — Figure S1: Cell types of PBMCs in a case and control pair. Cell types were annotated by SingleR using the celldex DatabaseImmuneCellExpressionData() reference [1]. Figure S2: Hallmark Overrepresentation Analysis for DE genes. Hallmark gene set overrepresentation analysis (ORA) were done using the clusterProfiler R package [24] and the msigdbr R package [7]. jgm70030‐sup‐0002‐supplementary.docx. [file JGM-27-e70030-s002.docx]

**Single-cell RNA Sequencing of Peripheral Blood Mononuclear Cells in Patients with Single Ventricle/Hypoplastic Left Heart Syndrome**

Hui-Qi Qu^1^, Kayleigh Ostberg^1^, Diana J Slater^1^, Fengxiang Wang^1^, James Snyder^1^, Cuiping Hou^1^, John J Connolly^1^, Michael March^1^, Joseph T Glessner^1,2,3^, Charlly Kao^1^, Hakon Hakonarson^1,2,3,4,5*^.

**Supplementary Materials**

**Supplementary Table 1 Average log2FC of the DE genes by the 15 cell types**

*Positive value means higher expression in cases.

**Supplementary Table 2 DE genes with opposite directions in different cell types**

*Positive value means higher expression in cases.

**Supplementary Table 3 The DE genes grouped into different modules**

**Supplementary Table 4 Significant Hallmark gene sets overrepresented in each module**


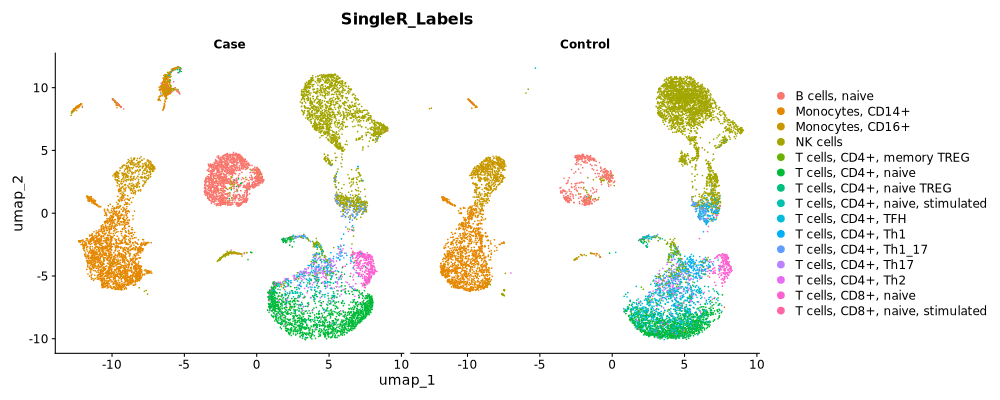


**Supplementary Figure 1** Cell types of PBMCs in a case and control pair. Cell types were annotated by SingleR using the celldex DatabaseImmuneCellExpressionData() reference[1].


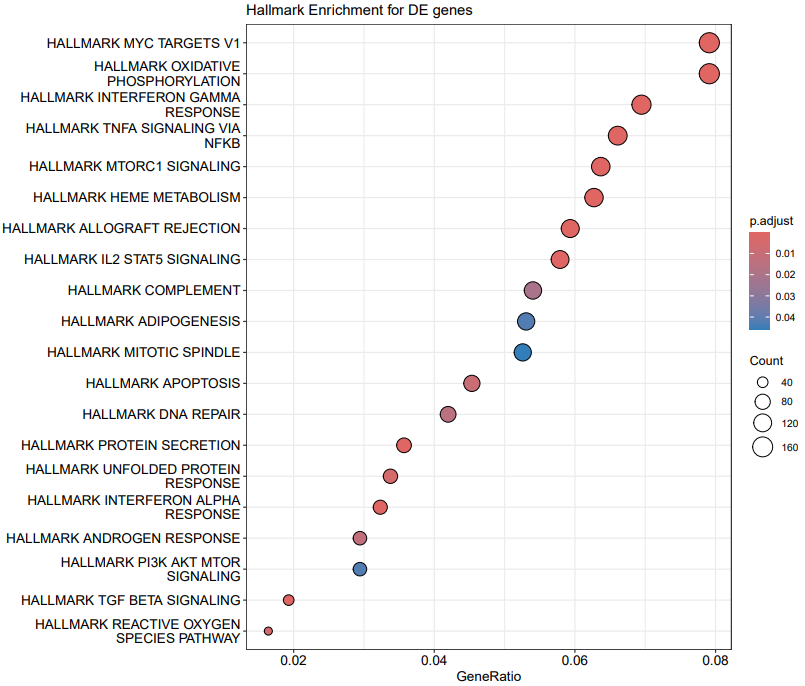


**Supplementary Figure 2** Hallmark Overrepresentation Analysis for DE genes. Hallmark gene set overrepresentation analysis (ORA) were done using the clusterProfiler R package[3] and the msigdbr R package[2].

**References**

1. Aran D, Looney AP, Liu L, Wu E, Fong V, Hsu A, Chak S, Naikawadi RP, Wolters PJ, Abate AR (2019) Reference-based analysis of lung single-cell sequencing reveals a transitional profibrotic macrophage. Nature immunology 20:163-172

2. Dolgalev I (2020) msigdbr: MSigDB gene sets for multiple organisms in a tidy data format. R package version 7

3. Yu G, Wang L-G, Han Y, He Q-Y (2012) clusterProfiler: an R package for comparing biological themes among gene clusters. Omics: a journal of integrative biology 16:284-287
